# A Retrospective Review of Neglected Tropical Diseases Diagnosed on Histopathological Specimens in the Free State Province, South Africa, 2015–2020

**DOI:** 10.1155/2024/5076288

**Published:** 2024-06-25

**Authors:** Danita Linda le Grange, She'neze Fatima Pillay, Liska Budding, Cornel van Rooyen, Jacqueline Goedhals

**Affiliations:** ^1^Department of Anatomical Pathology, School of Pathology, Faculty of Health Sciences, University of the Free State and National Health Laboratory Services, Bloemfontein, South Africa; ^2^Department of Chemical Pathology, School of Pathology, Faculty of Health Sciences, University of the Free State and National Health Laboratory Services, Bloemfontein, South Africa; ^3^Department of Biostatistics, School of Biomedical Sciences, Faculty of Health Sciences, University of the Free State, Bloemfontein, South Africa

## Abstract

**Background:**

Neglected tropical diseases (NTDs) are a heterogeneous group of medical conditions that commonly occur in underprivileged populations. NTDs are primarily diagnosed in tropical areas. Although South Africa is not situated in a tropical region, the high poverty rate makes the country susceptible to some NTDs. Limited data are available on the burden of NTDs in the Free State province of South Africa. This study aimed to determine the number of NTDs diagnosed on histopathological specimens in the public sector of the Free State province over a six-year period and to evaluate the patient demographics.

**Methods:**

A retrospective, descriptive study was performed. All NTDs diagnosed in histopathological specimens from public sector hospitals in the province submitted to the Department of Anatomical Pathology, National Health Laboratory Service, and University of the Free State between 1 January 2015 and 31 December 2020 were included in the study. The demographic information, biopsy site, and referring hospital were noted for each case identified.

**Results:**

A total of 72 NTDs were diagnosed. The five most common diagnoses were echinococcosis (*n* = 33; 45.8%), bilharzia (*n* = 13; 18.1%), leprosy (*n* = 9; 12.5%), mycetoma (*n* = 8; 11.1%), and intestinal worms (*n* = 5; 6.9%). Ten (30.3%) patients diagnosed with echinococcosis came from the Free State's neighbouring country, Lesotho.

**Conclusion:**

Echinococcosis was the most prevalent NTD diagnosed in central South Africa. We recommend that the South African Department of Health add echinococcosis to the principal NTDs of significance in South Africa, alongside soil-transmitted helminths, schistosomiasis, leprosy, and rabies.

## 1. Background

Approximately 20% of the global population is at risk of infection with neglected tropical diseases (NTDs) [1]. This percentage equates to a total disease burden of 1.6 billion people worldwide and the continent of Africa contributes 39% of this burden [2]. NTDs most frequently affect impoverished communities and contribute towards stigma, morbidity, and mortality [1].

The World Health Organization (WHO) recognises 20 diseases and their associated manifestations as NTDs [3, 4]. These include bacterial, viral, fungal, protozoal, and helminth-associated infections, as well as ectoparasitic infestations and noninfectious disorders [3]. Bacterial NTDs include leprosy (Hansen's disease), trachoma, yaws (endemic treponematoses), and Buruli ulcer. Rabies, dengue fever, and chikungunya comprise the viral NTDs. Mycetoma is considered both a bacterial and fungal NTD. Leishmaniasis, Chagas disease, and human African trypanosomiasis encompass neglected tropical parasitic infections. Neglected helminth-associated diseases include soil-transmitted intestinal helminthiases (ascariasis, trichuriasis, hookworm diseases, and strongyloidiasis), schistosomiasis, onchocerciasis, taeniasis/cysticercosis, lymphatic filariasis, echinococcosis, dracunculiasis, and foodborne trematodiases. Scabies and other ectoparasites are deemed to be neglected ectoparasitic tropical diseases. Snakebite envenomation is also regarded as a noninfectious neglected tropical condition [3].

The Department of Health of South Africa currently regards four principal NTDs of significance in South Africa: soil-transmitted helminths, schistosomiasis, leprosy, and rabies [5]. Both schistosomiasis and soil-transmitted helminths are amendable to preventative chemotherapy and are therefore of public health interest in South Africa [6]. Mass drug administration, in the form of school-based deworming programmes, has demonstrated efficacy in decreasing the burden of soil-transmitted helminths in children [7]. Statistical models suggest that mass drug administration should expand to include adults in deworming programmes to accomplish effective helminthic control [7, 8].

Schistosomiasis exhibits endemicity in 28 out of the 36 districts that have been delineated for endemicity assessment within South Africa. A total of 16 districts out of the 52 districts within the nation have yet to undergo the process of mapping and subsequent evaluation for their endemicity status. This parasitic ailment is primarily attributed to two species, namely, *Schistosoma haematobium* and *S. mansoni*, both of which are firmly entrenched in the South African environment [5].

Schistosomiasis can result in marked morbidity and long-term complications are a result of the chronic inflammatory response associated with trapped schistosome eggs. *S. haematobium* can result in urinary tract obstruction, while *S. mansoni* can lead to intestinal disease and liver fibrosis [9].

Another NTD of significance in South Africa is rabies [5]. Rabies is associated with a 99.9% fatality rate. Even though rabies is 100% vaccine-preventable, close to 60,000 deaths occur globally due to this disease [10]. Rabies is endemic in South Africa, but its incidence varies greatly between provinces [5]. Domestic dogs are the primary transmitter of rabies to humans, accounting for 85% of laboratory-confirmed rabies cases. This emphasises the importance of vaccinating domestic dogs against rabies [5].

Approximately 50 patients in South Africa are diagnosed annually with leprosy [5]. More than 75% of these cases are imported by cross-border entry from neighbouring countries. Leprosy has a high cure rate if detected and treated early. Patients in South Africa, however, often present late to healthcare facilities, resulting in decreased cure rates [5].

The evaluation of histopathological specimens can demonstrate the presence of infectious pathogens. Histological specimen evaluation is, therefore, critical in diagnosing NTDs and assisting medical practitioners in initiating appropriate therapy timeously [11]. At the time of conducting this retrospective review, no data were available on the burden of NTDs diagnosed on histological specimens from hospitals in the Free State province in South Africa [12]. The aim of this study was to determine the number of NTDs diagnosed on histopathological specimens submitted by public sector hospitals in the Free State province for the period of 1 January 2015–31 December 2020, and to evaluate patients' demographic characteristics.

## 2. Methods

A retrospective, descriptive study was conducted. All NTDs diagnosed on histopathological specimens received by the Department of Anatomical Pathology, University of the Free State (UFS), and National Health Laboratory Service (NHLS) from public sector hospitals in the Free State between 1 January 2015 and 31 December 2020 were included in the study, irrespective of the patients' residential location. Cases from hospitals outside the province were excluded. The cases were identified by performing Systematized Nomenclature of Medicine (SNOMED) key term searches on the NHLS laboratory information system for the 20 official NTDs, as recognised by the WHO [3]. The data collected from the pathology reports included the patients' age, gender, year of diagnosis, biopsy site, indication for biopsy, and final NTD diagnosis. The residential area of the patient and the name of the public sector hospital that submitted the sample were also recorded.

A pilot study of the first five cases was undertaken and the results were included in the final analysis. Descriptive statistics using frequencies were performed. Continuous variables were summarised by medians, minimum, maximum, or percentiles. Categorical variables were summarised by frequencies and percentages. The analysis was performed by the Department of Biostatistics, UFS, using SAS version 9.4 (SAS Institute Inc.; Cary, NC, USA).

The study was approved by the Health Sciences Research Ethics Committee (HSREC) of the University of the Free State (UFS) in Bloemfontein, South Africa (ethics approval reference no. UFS-HSD2021/1023/3108). Data were collected from archived laboratory specimens and no identifying particulars were used, hence no consent from patients was required.

## 3. Results

**Figure 1:**
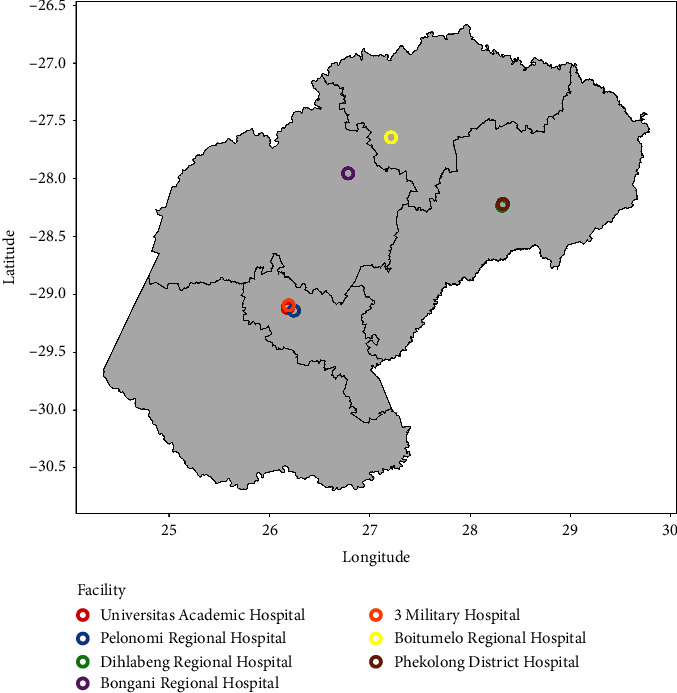
Map of facilities in the Free State province diagnosing NTDs.

Figure 1 represents a map of the Free State province showing the location of facilities in the province where NTDs could be diagnosed. A total of 74 patients were diagnosed with an NTD in the study period. Two of the 74 cases were excluded as they were submitted by hospitals in the Northern Cape province, giving a sample of 72 cases.

The patients had a median age of 27.5 years (range 3–64 years). Forty-two (58.3%) patients were male (Table 1). As summarised in Table 2, most specimens were submitted from hospitals in Bloemfontein, with 52 (72.2%) cases from Universitas Academic Hospital (UAH) and 12 (16.7%) cases from Pelonomi Tertiary Hospital.

The indications for biopsy varied. Regrettably, a comprehensive clinical indication and history were often not present on laboratory request forms. The most common indication specified on the request forms was mass lesions identified on imaging studies. Macroscopic haematuria and sandy patches on cystoscopy were the most common indications for biopsy for suspected urogenital schistosomiasis. A few NTD diagnoses were incidental after routine specimen submissions, such as specimens received for bilateral tubal ligation, a myomatous uterus, and cystic structures identified in the omentum during caesarean section.

The diagnoses are summarised in Table 3. The most common NTD identified was echinococcosis (*n* = 33; 45.8%), followed by bilharzia (*n* = 13; 18.1%), leprosy (*n* = 9; 12.5%), and mycetoma (*n* = 8; 11.1%). Different types of bilharzia and leprosy are distinguished in Table 3.

**Figure 2:**
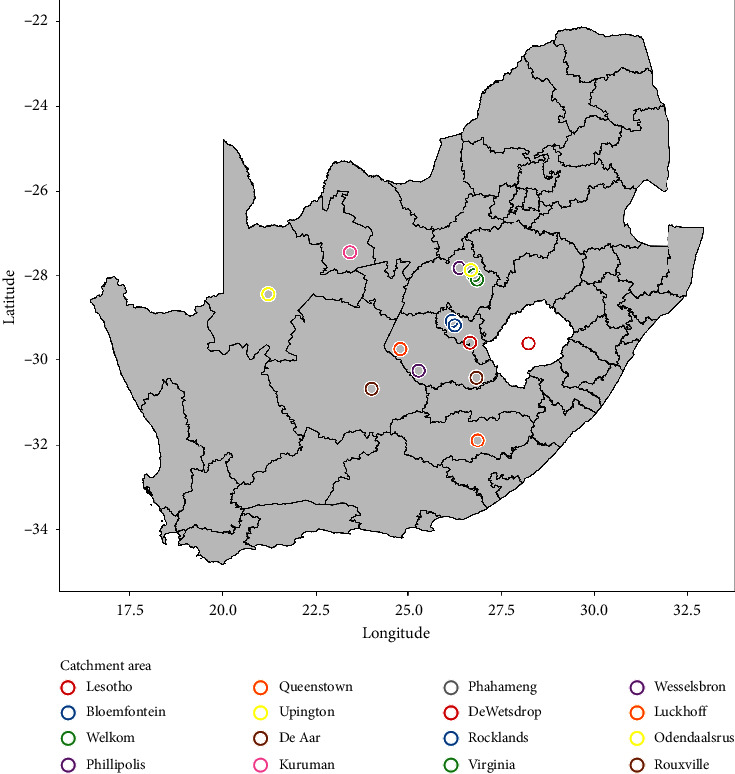
Map showing the origin or place of residence of patients diagnosed with echinococcosis.

Table 4 lists the biopsy sites from which echinococcosis was diagnosed. The majority of *Echinococcus* infestations occurred in the lungs (*n* = 17; 51.5%), followed by the brain (*n* = 7; 21.2%) and bone (15.2%). Most patients diagnosed with echinococcosis (*n* = 10; 30.3%) came from Lesotho, a small country neighbouring South Africa, followed by Bloemfontein (*n* = 4; 12.1%) and Welkom (*n* = 3; 9.1%). The remainder of the cases was identified intermittently throughout the Free State. Patients residing in the Northern Cape (*n* = 3; 9.1%) and Eastern Cape (*n* = 1; 3.0%) provinces, and two patients from unknown locations, were also diagnosed with echinococcosis based on specimens received from hospitals in the Free State. *Ascaris lumbricoides* was the only intestinal parasite identified in this study. In Figure 2, a map of South Africa shows the origin or place of residence of patients diagnosed with echinococcosis.

## 4. Discussion

According to the WHO document on ending the neglect to attain the Sustainable Development Goals 2021–2030, critical action is required to control and/or eradicate the following NTDs: echinococcosis, foodborne trematodiases, leishmaniasis, mycetoma, chromoblastomycosis, scabies, snakebite envenomation, cysticercosis, rabies, and yaws, listed in no particular order [13].

### 4.1. Echinococcosis

Echinococcosis is a parasitic tapeworm disease belonging to the genus *Echinococcus*. Echinococcosis is subdivided into alveolar, cystic, or neotropical echinococcosis, of which only alveolar and cystic echinococcosis are of medical importance [14]. For the purpose of this study, we will only discuss cystic echinococcosis, as it was the only form identified in central South Africa.

Cystic echinococcosis, also known as hydatid disease or hydatidosis, is caused by *Echinococcus granulosus* [14]. *Echinococcus* cycles between two hosts, usually domestic dogs (definitive hosts) and herbivores (intermediate hosts). Humans are accidental intermediate hosts and become infested following the consumption of parasite eggs in food or water contaminated by dog faeces. Cystic echinococcosis is endemic on every continent on the globe, except Antarctica. The incidence of cystic echinococcosis is highest in rural areas [14].

The liver has been identified as the most common site of infection of cystic echinococcosis, followed by the lungs. Less commonly, cysts may also occur in the kidneys, spleen, heart, bone, and central nervous system [15]. Our study identified the lung as the most common site of infection, followed by the brain, which was noteworthy, as the brain is considered an “infrequent” site of parasitic infection in the disease's endemic and nonendemic regions.

In this study, most echinococcosis cases were imported from Lesotho. In other words, patients from Lesotho were diagnosed and treated at Free State hospitals, particularly UAH. Lesotho is a neighbouring country of South Africa. It is situated on the southeastern border of the Free State province. Health care resources are extremely limited in Lesotho and this may explain why patients were referred to UAH in South Africa. Patients may have been referred to UAH for specialised care because clinicians in resource-restricted facilities in Lesotho are unable to conduct specialised examinations or biopsy techniques, for example, brain biopsies, lung biopsies, or ultrasound-guided liver biopsies.

### 4.2. Schistosomiasis

Schistosomiasis or bilharzia is a parasitic disease transmitted through contact with cercariae-contaminated freshwater [16]. Schistosomiasis is classified into two groups, each associated with a particular causative pathogen: intestinal and urinary schistosomiasis. *Schistosoma mansoni* and *S. japonicum* cause intestinal schistosomiasis, while *S. haematobium* causes urinary schistosomiasis. Disease associated with other species occurs less frequently [16].

Schistosomiasis is the second most common parasitic disease in Africa, after malaria [16]. Approximately, 240 million people are affected by schistosomiasis worldwide, with more than 700 million people residing in endemic regions [17]. The prevalence of schistosomiasis in children residing in some endemic areas of South Africa is estimated to be 95%. Endemic areas in South Africa include Limpopo, Mpumalanga, Gauteng, KwaZulu-Natal, and Eastern Cape provinces [17].

Due to the hot and dry climate in central South Africa, the prevalence of schistosomiasis is not as high as in the tropical and subtropical regions. A possible explanation for our data could be that the cases diagnosed in central South Africa might be related to travel from one of the endemic regions. We are, however, unable to validate this argument due to inadequate clinical information provided on the laboratory specimen request forms.

### 4.3. Leprosy

Leprosy, or Hansen's disease, is a notifiable infectious, contagious disease caused by *Mycobacterium leprae*. This disease mainly affects the integumentary system, peripheral nervous system, eyes, and mucosal surfaces of the upper respiratory tract [18]. Transmission of leprosy is not well understood. It is postulated that droplet spread and prolonged contact with infectious persons may enhance the spread of leprosy [19].

Leprosy is considered a curable disease, and disability may be avoided if detected and treated early [18, 19]. The WHO recommends a multidrug therapy (MDT) regimen consisting of dapsone, rifampicin, and clofazimine for six months for paucibacillary leprosy and 12 months for multibacillary leprosy. This MDT regimen has proven effective in eliminating the pathogen and curing the patient. The WHO has provided free access to the regimen through the Nippon Foundation since 1995 [18].

Annually, approximately 50 patients in South Africa are diagnosed with leprosy, with most cases entering from neighbouring countries [5]. Even though leprosy is a curable disease, patients in South Africa often present late to healthcare facilities. Late presentation is associated with low cure rates. This is a common occurrence in South Africa due to stigma, limited awareness in the community, and lack of knowledge concerning the current clinical diagnosis of leprosy [5].

Although borderline leprosy is the most common form of leprosy, our study demonstrated most leprosy cases to be paucibacillary. The WHO classifies paucibacillary leprosy as a single skin lesion with severe peripheral nerve involvement [20]. We could not discern the clinical presentation or duration of symptoms due to insufficient information provided on laboratory forms.

### 4.4. Mycetoma

Mycetoma or “Madura foot” is a chronic infectious disease caused by microorganisms, most often by bacteria (*Actinomyces* and *Nocardia*). Causal fungal organisms include *Madurella* and *Pseudallescheria*. This disease leads to the progressive destruction of subcutaneous tissues involving the integumentary, muscular, and skeletal systems [21–24].

The foot is the body part most commonly affected by mycetomas [21–24]. Our findings concurred with previously published studies. Of the eight cases of mycetoma identified in our study, 62.5% (*n* = 5) of cases diagnosed on histopathological sections involved the foot as the primary site of infection. Swelling with pain, sinus tract formation, and yellow granules in exudate characterise mycetomas [21]. Transmission of mycetoma occurs via the traumatic inoculation of causative organisms into subcutaneous tissues [22, 23].

Mycetoma occurs more commonly in people who live in rural areas and walk barefoot [21]. Mycetoma infections occur more frequently in these regions located in the “mycetoma belt,” an area that spans between the latitudes of 15 degrees south and 30 degrees north of the equator [23]. Although South Africa is not situated in the “mycetoma belt,” mycetomas were identified as the fourth most prevalent NTD in our study.

### 4.5. Intestinal Worms

Intestinal worms were identified as the fifth most prevalent NTD diagnosed on histopathological specimens in the Free State. *Ascaris lumbricoides* was the only intestinal helminth identified in this study. Histopathological specimens yielding *Ascaris* parasites and ova included specimens from the gastrointestinal tract and the placenta. *Ascaris* worms can be identified macroscopically in gastric contents and stool samples. Although these specimens are usually evaluated by microbiologists, two such cases containing *Ascaris* parasites were submitted to the Department of Anatomical Pathology. *Ascaris* parasites were also, surprisingly, noted on the amniotic membrane of the placenta submitted for histopathological evaluation. In this case, the worms most likely represented faecal contamination of the specimen. *Ascaris* ova were identified within the appendix.

*Ascaris*, and some other nematode worms, forms part of the group of soil-transmitted helminths [7]. They may either present asymptomatically or result in considerable morbidity. Heavy infections are associated with gastrointestinal symptoms, anaemia, and impaired physical and cognitive development in children [7]. Our study demonstrated the most common reason for presentation to be gastrointestinal complaints, such as worms passed in stool or vomitus. We expected ascariasis to be the most common helminth, as it accounts for 70% of endemic helminth species in South Africa [5]. Morbidity control programs are centred on the mass administration of anthelminthic drugs to school-going children and other risk groups [7].

### 4.6. Cysticercosis

Cysticercosis is a parasitic infection caused by the larval stage of the intestinal tapeworms *Taenia solium*, *T. saginata,* and *T. asiatica* [25]. Infections with *T. saginata* and *T. asiatica* are caused by consuming infected and inadequately cooked beef or pig liver, respectively. Infection with *T. solium* results from the consumption of infected and inadequately cooked pork or through autoinfection. *T. solium* is considered the only species with a significant health impact [25].

The larvae of cysticercosis may infect the muscular system, integumentary system, eyes, and central nervous system. When the central nervous system is affected, it is known as neurocysticercosis and may result in epilepsy. Cysticercosis is responsible for 30% of epilepsy cases in endemic regions. Treatment is by anthelminthic agents such as praziquantel and albendazole, or occasionally surgery [25].

Our study demonstrated neurocysticercosis to be the most common presentation, with cysts found in either the brain or spinal column. It is unknown if these patients suffered from epilepsy, as complete clinical histories were not provided.

### 4.7. Scabies

Scabies is a parasitic dermatological disease caused by *Sarcoptes scabiei* var. *hominis*. It occurs worldwide, and millions of people may be affected at any given time. Transmission is by contact with an infected person or fomites, and treatment is by topical scabicides [26].

### 4.8. Limitations

The limited number of cases identified in our study (*n* = 72) was expected, as central South Africa has grassland and savanna-type vegetation compared to the tropical and subtropical regions where NTDs prevail. In addition, COVID-19 has had a vast impact on the surveillance, diagnosis, treatment, and prevention programs of NTDs because attention had been focused on combating the pandemic [27]. Although this study spanned a period of six years, we noticed that in 2020, only 6.9% (*n* = 5) of the NTDs had been identified. This may be attributed to travel restrictions imposed by the South African government during the 2020 lockdown. In 2020, no NTDs were diagnosed in patients from Lesotho, a country that was identified as a large contributor to the burden of NTDs in the Free State. This might have resulted in the underdiagnosis of NTDs in our study. Due to COVID-19-related anxiety and fear of contracting the virus in the hospital setting, many patients with NTDs might also not have sought medical care.

## 5. Conclusion

The majority of NTDs in our study were caused by helminths. Collectively, echinococcosis, bilharzia, cysticercosis, and intestinal worms contributed 75.0% (*n* = 54) of the NTD cases diagnosed in public sector hospitals in the Free State province. NTDs are indeed neglected, as proven by the lack of data available from the literature. We could not identify any similar studies conducted on NTDs in South Africa. We suspect that NTDs are underreported and underdiagnosed. Considering this lack of epidemiological information on NTDs in South Africa, we identified the need to conduct further studies to update current data on these neglected but important diseases.

## Figures and Tables

**Table 1:** Sex distribution in NTDs diagnosed.

Sex	*n* (%)
Female	30 (41.7)
Male	42 (58.3)

**Table 2:** Number of specimens received per hospital.

Hospital (town/city)	*n* (%)
Universitas Academic Hospital (Bloemfontein)	52 (72.2)
Pelonomi Regional Hospital (Bloemfontein)	12 (16.7)
Dihlabeng Regional Hospital (Bethlehem)	3 (4.2)
Bongani Regional Hospital (Welkom)	2 (2.8)
3 Military Hospital (Bloemfontein)	1 (1.4)
Boitumelo Regional Hospital (Kroonstad)	1 (1.4)
Phekolong District Hospital (Bethlehem)	1 (1.4)
Total	72 (100)

**Table 3:** Diagnoses of neglected tropical diseases.

Diagnosis	*n* (%)
Echinococcosis	33 (45.8)
Bilharzia	13 (18.1)
Urogenital	11 (84.6)
Intestinal	2 (15.4)
Leprosy	9 (12.5)
Paucibacillary	6 (66.7)
Multibacillary	2 (22.2)
Indeterminate	1 (11.1)
Mycetoma	8 (11.1)
Intestinal worms^*∗*^	5 (6.9)
Cysticercosis	3 (4.2)
Scabies	1 (1.4)
Total	72 (100)

^*∗*^Although ascariasis, trichuriasis, hookworm diseases, and strongyloidiasis were considered in this study, ascaris lumbricoides was the only soil-transmitted helminth identified.

**Table 4:** Biopsy site from which the diagnosis of echinococcosis (*n* = 33) was made.

Biopsy site	*n* (%)
Lung	17 (51.5)
Brain	7 (21.2)
Bone	5 (15.2)
Liver	2 (6.1)
Omentum	1 (3.0)
Mediastinum	1 (3.0)

## Data Availability

The data used to support the findings of this study are available from the corresponding author upon request.
